# Sweet Shop Sialagogues: A Sour Solution to Sialolithiasis

**DOI:** 10.7759/cureus.32097

**Published:** 2022-12-01

**Authors:** Simon Morris, Jake Ahmed, Simon Browning

**Affiliations:** 1 Otolaryngology, Betsi Cadwaladr University Health Board, Bangor, GBR; 2 Otolaryngology, Swansea Bay University Health Board, Baglan, GBR

**Keywords:** otolaryngology, sialagogue, citrus, salivary gland calculi, saliva

## Abstract

Historically, boiled sweets have been recommended by ear, nose, and throat surgeons for their sialagogue effect in patients with sialolithiasis. This study presents an in vivo analysis of boiled sweets and solutions to determine sialagogue superiority. Six high-street boiled sweets (lemon sherbets, rhubarb sweets, mint humbugs, Werther’s Original® (August Storck, Germany), Fox's Glacier Fruits® (Fox's Confectionery, Braunstone, Leicester, United Kingdom), and Chupa Chups® lollipops (Perfetti Van Melle, Breda, Netherlands)) and two solutions (malt vinegar and lemon juice) were compared to two controls (no sweet and inert plastic) in two healthy participants. Malt vinegar and lemon juice produced the highest salivary flow. The best-performing boiled sweets were Chupa Chups lollipops and lemon sherbet. Substances with the highest concentrations of citric or lactic acid were the best sialagogues. This pilot study provides a proof of concept for further investigation in this cohort of patients.

## Introduction

Sialolithiasis involves the formation of stones within the ducts of major salivary glands of the head and neck [1]. There are a variety of presenting features resulting from this obstruction, which include recurrent prandial discomfort, acute sialadenitis, and abscess formation. Sialolithiasis is estimated to affect one in 15,000 people in the United Kingdom (UK) [2].

Management of sialolithiasis typically starts with conservative measures such as analgesia, warm compresses, salivary gland massage, and sialagogues [3]. Literature has examined the use of various substances such as pilocarpine and other parasympathomimetics, where the sialagogue effect is theorised to promote salivation, increase salivary secretion, and allow progression of the stone and its spontaneous expression from the papilla [4,5].

Historically, lemon drops, due to their sour and citric essences, have been recommended by clinicians for their sialagogue effect. However, to our knowledge, no study has previously investigated the sialagogue effect of other common high-street sweets or solutions to determine sialagogue superiority.

Salivary secretion has two primary phases, notably, the cephalic (the sight or smell of food) and buccal phases (taste of food in mouth) [6,7]. In an adult, the average flow of saliva varies between 1 litre and 1.5 litres per day, and the acceptance rate for unstimulated salivary flow is approximately 0.3 millilitres (ml) per minute [8,9].

This novel in vivo pilot study aims to determine which common high-street substances have the superior sialagogue effect, as a potential adjuvant therapy for conservative management of patients with sialolithiasis.

## Materials and methods

Two healthy participants (the co-authors) with no oral or salivary gland disease were test subjects.

Six common UK high-street boiled sweets and two sour acidic liquid preparations were selected and compared to two control measures. The six sweets were lemon sherbets, rhubarb sweets, mint humbugs, Werther’s Original® (August Storck, Germany), Fox's Glacier Fruits® (Fox's Confectionery, Braunstone, Leicester, United Kingdom), and Chupa Chups® lollipops (Perfetti Van Melle, Breda, Netherlands). The two solutions were malt vinegar and pure lemon juice. The two control items were ‘no sweet’ and an inert piece of plastic.

The test protocol involved three phases, lasting a total of five minutes, where subjects were asked to collect their own saliva in a sterile plastic gallipot with measurements. Phase 1 included a one-minute pre-oral phase, where participants collected saliva production whilst looking at and smelling the test substance. Phase 2 followed with a two-minute oral phase, where participants collected their saliva whilst holding the substance in their mouths. Finally, phase 3 included a two-minute post-oral phase where subjects collected further saliva production following removal of the substance from their mouths. Where liquid stimuli were tested, 3ml of solution was used and subtracted from the final saliva production volume. Boiled sweets were used as bought and used sequentially without replenishment between each repeat.

This protocol was repeated three times by each participant for each test sweet and compared to the control.

Participants were asked not to look at or think about any other food items throughout testing to avoid the bias of contaminated thoughts. Testing took place at least 30 minutes following any other oral ingestion, and there was at least five minutes between each round of substance testing. 

Analysis

Anonymised data were entered into Microsoft Excel (Microsoft Corporation, Redmond, Washington, United States). Data are presented in millilitres (ml), with mean and range of saliva measurements to compare to existing literature.

Ethical considerations

Ethical approval was not considered necessary for this project. Both study participants were the consenting co-authors.

## Results

Two healthy participants took part. Both were male. The mean age was 30.5 years (range 27-34 years). All test substances promoted more salivary flow than the control items (no sweet and inert plastic).

**Figure 1:**
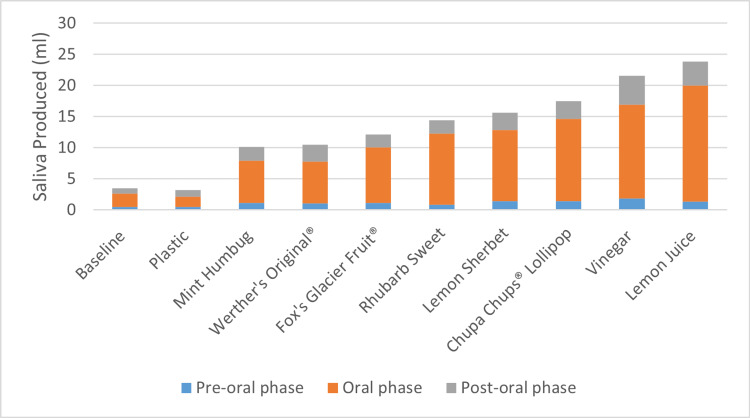
Graphical Representation of Total Saliva Production in Five Minutes According to the Test Item (Control, Sweet, or Solution)

**Figure 2:**
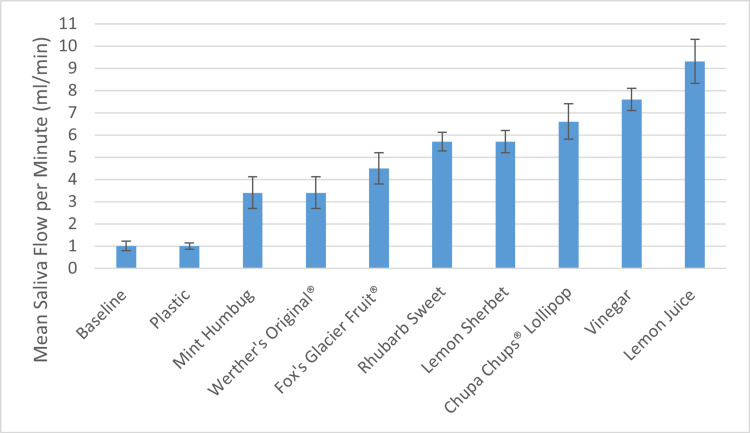
Mean (+/- Standard Deviation) Saliva Flow per Minute (ml/min) According to the Test Item and Reference to Adult Baseline Saliva Production

Malt vinegar and pure lemon juice produced a mean of 21.5ml and 23.8ml saliva, respectively, in the five-minute testing period (Figure 1). When considering salivary production based on the oral phase of testing, these two solutions promoted a rate of 7.4ml and 9.3ml of saliva per minute respectively (Figure 2). This is approximately nine times more than the control item.

The three best performing boiled sweets for salivary flow in the five-minute test period were Chupa Chups lollipops (17.5ml), lemon sherbet (15.7ml), and rhubarb sweets (14.4ml).

## Discussion

This study has shown that malt vinegar and pure lemon juice were clearly the superior sialagogues. Despite previous anecdotal evidence regarding boiled sweets, the two acidic solutions promoted the highest rates of salivary flow in this study. This occurred not only during the oral phase of the testing, but also the post-oral phase, indicating a prolonged effect of these two solutions as sialagogues. They promoted over nine times more saliva collection than when compared to the control and 25 times more than the baseline adult salivary production [6,7].

Sialagogues can be divided into peripheral sialagogues that stimulate a gustatory response, such as the test items described in this study, and central sialagogues that include parasympathomimetics such as pilocarpine [10]. Both types have multiple uses in the clinical setting by increasing secretions and diminishing complaints of sialadenitis or dry mouth in patients. However, the central-acting sialagogues are associated with greater side effects due to the non-selective muscarinic agonist properties [10-12].

This study has also demonstrated the positive sialagogue effects of various sweets commonly found in the UK. Despite historical opinion, lemon sherbets were not the superior sialagogue [13-15]. In this study, a common high-street brand of fruit lollipop was responsible for the highest salivary flow in the five-minute test period. When considering the ingredients of these boiled sweets, those that contained lactic or citric acid (lemon sherbets, fruit drops, and Chupa Chups lollipops) outperformed those that were milk-based (Werther’s Original and mint humbugs).

Anecdotally, the participants’ satisfaction in consuming the boiled sweets was far higher than when consuming the malt vinegar and lemon juice. Yet this did not impact the cephalic phase of the testing, which suggests that enjoyment of sweet does not necessarily correspond with increased salivation. However, the authors recognise that consumption of large quantities of vinegar or lemon juice may not present an attractive management option for patients with sialolithiasis. Likewise, it is important to highlight that the test substances used in this study were all high in sugars and may pose a potential risk of dental caries if consumed in excessive quantities.

Management of sialadenitis, xerostomia, and hyposalivation can be challenging [10]. This study aims to describe useful and freely available sialagogues that can be recommended by clinicians. The test items were chosen according to their frequency at local supermarkets and thus ease of availability for purchase (for both the authors’ and future patients’ convenience).

This pilot study has demonstrated the benefits of common high-street substances as sialagogues. Full statistical analysis has not been conducted owing to the small sample size. However, this pilot study acts as a proof of concept for further study to investigate the exact role of these substances in patients with sialolithiasis or xerostomia which the authors are planning with a larger sample size.

## Conclusions

This in vivo study highlights the value of malt vinegar, lemon juice, and various boiled sweets for sialagogue effects and the potential implications for the management of sialolithiasis or xerostomia. Contrary to previous beliefs, lemon sherbets were not the superior sialagogue; however, the substances with the highest concentrations of citric or lactic acid were the best performers in testing.
